# The risk factors associated with post-transplantation BKPyV nephropathy and BKPyV DNAemia: a prospective study in kidney transplant recipients

**DOI:** 10.1186/s12879-024-09093-7

**Published:** 2024-02-22

**Authors:** Camilla Lorant, Justina Zigmantaviciute, Naima Ali, Ursa Bonnevier, Mattias Tejde, Bengt von Zur-Mühlen, Britt-Marie Eriksson, Anders Bergqvist, Gabriel Westman

**Affiliations:** 1 Department of Medical Sciences, Section of Infectious Diseases, Uppsala University, SE-751 85 Uppsala, Sweden; 2 Department of Medical Sciences, Clinical Microbiology, Uppsala University, Uppsala, Sweden; 3 Clinical Microbiology and Infection Control, Uppsala University Hospital, Uppsala, Sweden; 4 Department of Nephrology, Gävle Hospital, Gävle, Sweden; 5 Department of Nephrology, Falun Hospital, Falun, Sweden; 6 Department of Surgical Sciences, Section of Transplantation Surgery, Uppsala University, Uppsala, Sweden

**Keywords:** BK Polyomavirus (BKPyV), BKPyV-associated nephropathy (BKPyVAN), BKPyV genotype, BKPyV risk factors, Kidney transplantation, Immunosuppression

## Abstract

**Background:**

BK polyomavirus (BKPyV) infection after kidney transplantation can lead to serious complications such as BKPyV-associated nephropathy (BKPyVAN) and graft loss. The aim of this study was to investigate the incidence of BKPyVAN after implementing a BKPyV screening program, to map the distribution of BKPyV genotypes and subtypes in the Uppsala-Örebro region and to identify host and viral risk factors for clinically significant events.

**Methods:**

This single-center prospective cohort study included kidney transplant patients aged ≥ 18 years at the Uppsala University Hospital in Sweden between 2016 and 2018. BKPyV DNA was analyzed in plasma and urine every 3 months until 18 months after transplantation. Also genotype and subtype were determined. A logistic regression model was used to analyze selected risk factors including recipient sex and age, AB0 incompatibility and rejection treatment prior to BKPyVAN or high-level BKPyV DNAemia.

**Results:**

In total, 205 patients were included. Of these, 151 (73.7%) followed the screening protocol with 6 plasma samples, while184 (89.8%) were sampled at least 5 times. Ten (4.9%) patients developed biopsy confirmed BKPyVAN and 33 (16.1%) patients met criteria for high-level BKPyV DNAemia. Male sex (OR 2.85, *p* = 0.025) and age (OR 1.03 per year, *p* = 0.020) were identified as significant risk factors for developing BKPyVAN or high-level BKPyV DNAemia. BKPyVAN was associated with increased viral load at 3 months post transplantation (82,000 vs. < 400 copies/mL; *p* = 0.0029) and with transient, high-level DNAemia (*n* = 7 (27%); *p* < 0.0001). The most common genotypes were subtype Ib2 (*n* = 50 (65.8%)) and IVc2 (*n* = 20 (26.3%)).

**Conclusions:**

Male sex and increasing age are related to an increased risk of BKPyVAN or high-level BKPyV DNAemia. BKPyVAN is associated with transient, high-level DNAemia but no differences related to viral genotype were detected.

**Supplementary Information:**

The online version contains supplementary material available at 10.1186/s12879-024-09093-7.

## Background

BK polyomavirus (BKPyV) often causes asymptomatic infection during childhood [1]. The seroprevalence has been examined in different populations using various approaches [2] and recent studies in healthy adults and recipients of organ transplants estimate a seroprevalence above 90% [3–6]. After primary infection, BKPyV persists mainly in the urothelium and renal tubular cells in the reno-urinary tract causing minimal clinical implications [7]. However, in immunocompromised hosts BKPyV may reactivate with detectable BKPyV DNA in urine and plasma and cause serious complications such as BKPyV-associated nephropathy (BKPyVAN) in 1–10% of kidney transplant recipients [1, 8–11]. Besides BKPyV DNAemia, several other risk factors have been identified, including male sex, older age, concurrent cytomegalovirus (CMV) infection, HLA mismatch, AB0 incompatibility, rejection treatment and deceased donor [12–15].

BKPyV is classified into four genotypes labelled I-IV [2, 16]. Genotype I is the most common variant (80%) and is prevalent worldwide. Genotype IV is less frequent (15%) and is mostly found in Europe and Asia, whereas genotypes II and III are rare [17, 18]. Based on variations in the BKPyV VP1 gene, four genotype I subtypes (Ia, Ib1, Ib2, and Ic) and six genotype IV subtypes (IVa1, IVa2, IVb1, IVb2, IVc1, and IVc2) have been identified [18, 19]. Subtype Ia is highly prevalent in Africa, Ib and Ic in Southeast and Northeast Asia respectively and Ib2 is highly prevalent in Europe [18, 20]. Subtypes belonging to genotype IV are predominantly observed in Asia, except for IVc2, which is more prevalent in Europe [19, 21]. Based on in vitro experiments, BKPyV genotype I replicates more efficiently than genotype IV in human renal epithelial cells in vitro [22] and therefore could be more capable to cause clinically relevant BKPyV infection. Some studies have shown that genotype IV is associated with higher DNAemia and BKPyVAN [23] while others have not been able to verify this observation [24] and a clear correlation between genotype and clinical outcome of BKPyV infection has not yet been established.

In a previous retrospective study of 928 renal transplants, we found that male sex was the only statistically significant predictor for BKPyVAN [25]. In 2015, a screening program for BKPyV was introduced at our center. In this study we evaluated the incidence of BKPyVAN after implementation of the program. We also investigated selected pre-transplant risk factors for BKPyVAN and levels of BKPyV DNAemia as well as post-transplant variables such as DNA levels and BKPyV genotypes that were only related to BKPyVAN.

## Methods

### Study design

This was a single-center prospective cohort study. The study included females and males aged ≥ 18 years who underwent kidney transplantation or simultaneous pancreas and kidney transplantation at the Uppsala University Hospital in Sweden from 12th of May 2016 until the 24th of September 2018 and signed the informed consent (inclusion criteria). Patients who did not understand sufficient Swedish, refused, had psychiatric problems or failed to follow-up were not able to participate (exclusion criteria). The study was approved by the Regional ethical review board in Uppsala (No. 2015/488). Data were collected from electronic health records and the local transplantation database at the Uppsala University Hospital.

### Local BKPyV screening program

A screening program for BKPyV was implemented at our center during 2015 including analyses for BKPyV DNA in blood samples drawn approximately three months after transplantation and then approximately every third month until 18 months after transplantation. In addition, blood samples could also be taken for BKPyV DNA analysis in case of increased creatinine levels and/or suspected BKPyV infection. If BKPyV levels were elevated in plasma the patient was monitored with more frequent sampling. The study protocol included analysis of plasma samples close to all six time points (+/- 6 weeks). In addition to the mandatory study protocol, urine samples were taken up to 6 times, at the same time points as the plasma samples.

### Diagnosis of BKPyV and BKPyVAN

BKPyV was analyzed in plasma and urine samples from the patients using a modified variant of a previously described quantitative TaqMan real time polymerase chain reaction (qPCR) procedure, where the primers and probe were designed to give representative detection of all major genotypes [26] (Supplementary Table 1). Briefly, BKPyV DNA was extracted from 200 µL plasma or urine using the automatic NucliSens easyMAG robot (BioMérieux, Marcy l’Etoile, France). BKPyV DNA was then amplified from 5 µl out of 60 µl elution volume using TaqMan Universal PCR Master Mix (Thermo Fisher, Stockholm, Sweden) and the Qiagen Rotor-Gene Q thermo cycler (Qiagen, Hilden, Germany). The assay has a linear range of 400 to 1 × 10^9^ copies/mL and its proficiency was verified using external quality assessment programs from Instand and QCMD.

The locally applied indication for a transplant biopsy was, in general, an unexplained increase in serum creatinine of at least ten per cent. BKPyVAN was examined by pathological evaluation of kidney allograft biopsies taken at any time after transplantation and defined as positive immunohistochemical staining for Simian virus 40 large T antigen and a positive BKPyV DNAemia (≥400 copies/mL).

### Determination of BKPyV genotype

BKPyV genotypes were determined by targeting the variable region of the VP1 gene using Sanger sequencing and gene analysis in MEGA X [27]. The sequences were aligned using Clustal W and a maximum-likelihood phylogenetic tree was constructed based on the Tamura-Nei substitution model using default parameters. Viral DNA from plasma and urine samples was amplified using a nested PCR covering nucleotides 1528–2270 (Dunlop numbering) of the BKPyV genome (Supplementary Table 1), in a Veriti 96 well thermal cycler (Applied Biosystems) by Taq PCR master mix (QIAGEN).

### Immunosuppressive regimens

With few exceptions, patients received the local standard immunosuppressive regimen used during the study period; consisting of induction therapy with anti-IL-2 receptor antibodies (basiliximab) and methylprednisolone or methylprednisolone alone which was considered standard of care (SOC) in Uppsala at the time of the study. The maintenance immunosuppression consisted of daily tacrolimus and prednisolone in tapering doses. In addition, most patients received mycophenolate mofetil (MMF).

Enhanced induction was defined as treatment with thymoglobulin, rituximab and/or eculizumab, often in combination with IVIg (intravenous immunoglobulin) and immunoadsorption and/or plasmapheresis/apheresis. Enhanced induction was given on certain occasions such as HLA-incompatibility, AB0 incompatibility, simultaneous pancreas and kidney transplantation and other higher risk immunological scenarios such as previous transplantations. The standard of care induction for AB0 incompatibility was rituximab and glycosorb treatment or in some cases immunoadsorption and/or plasmapheresis. Rejection treatment included methylprednisolone, anti-thymocyte immunoglobulin, rituximab, eculizumab, IVIg and/or plasmapheresis.

The locally applied recommendation in case of BKPyV DNAemia detection with levels < 10,000 copies/mL was to increase follow-up to monthly plasma DNA analysis and make minor adjustments of the immunosuppressive treatment. If high-level DNAemia was detected, follow-up was increased and the MMF dose was reduced by 50%. If reduction of the MMF dose was inefficient MMF was discontinued. In most cases the tacrolimus dose was also reduced.

### Statistical analysis

The primary analysis was the incidence of BKPyVAN after implementation of a screening program. The secondary analysis was on predictors for BKPyVAN or high-level BKPyV DNAemia. The preselected risk factors in the multivariable analysis were male sex, age, AB0 incompability and rejection treatment, which were selected on the basis of previous findings and biological rationale rather than univariate analysis, in line with the recommendations by Heinze et al. [28]. As an exploratory analysis, the impact of BKPyV genotypes and virologic course on clinical outcome was investigated.

Quantitatively determinable levels of BKPyV DNA were categorized into the following groups: (a) High-level BKPyV DNAemia defined as levels of BKPyV DNA ≥10,000 copies/mL, which is considered to be a clinically relevant level to develop BKPyVAN [29, 30]; (b) Low-level DNAemia 400 − 10,000 copies/mL; (c) Positive in urine only.

A logistic regression model, with Firth’s method to handle the sparsity in the data, was used for evaluating the primary endpoint in relation to the risk factors. Comparisons of subgroups were performed using Fisher’s exact test, with multiplicity correction using the false discovery rate (FDR) method or by multiple Mann-Whitney U tests with Holm-Sidak correction.

Kidney function was evaluated with a mixed-effects model.

Continuous data were presented as median with ranges. Statistical analyses were conducted in R version 3.5.1 using package *survival* version 2.42-3 or GraphPad Prism version 9.5.1. *P*-values below 0.05 were considered significant.

## Results

### Study subjects

**Fig. 1:**
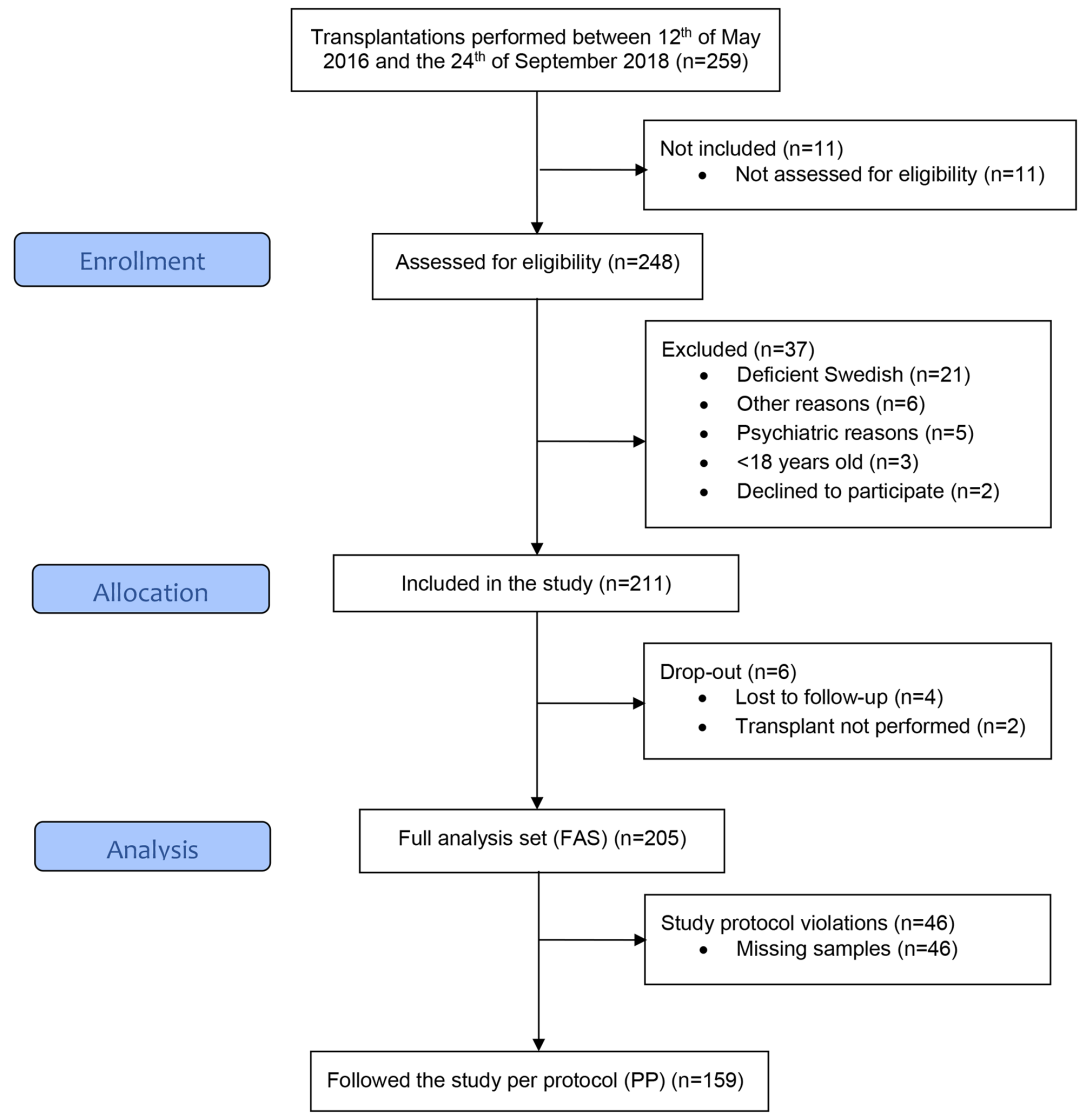
Flow chart of transplant patients in the study

In total, 259 patients were planned for transplantation during the study period, whereof 248 were assessed for eligibility. Of these, 37 patients were excluded due to not being expected to understand the spoken and written information (*n* = 21), psychiatric reasons (*n* = 5), being minors (*n* = 3), declined to participate (*n* = 2) or other reasons (*n* = 6). Hence 211 patients were included in the study of which 6 patients were later excluded because they were lost to follow-up (*n* = 4) or because the transplantation was not performed (*n* = 2). For a summary of the clinical setup, see Fig. 1.

The final cohort of 205 patients was defined as the full analysis set (FAS) and included all transplanted patients with any number of plasma samples taken. Of these, 159 patients followed the study per protocol (PP) with six plasma samples taken approximately every third month until the end of the observation period or until they met the primary endpoint, lost their graft or died within 18 months (±2 months) from the transplantation. Whereas 151 (73.7%) of the FAS subjects followed the screening program as intended with 6 plasma samples, 5 plasma samples were drawn from 33 patients, resulting in 184 (89.8%) with at least 5 samples. Patient characteristics are presented in Table 1.

**Table 1:** Patient characteristics

	FAS (n = 205)	FAS BKPyVAN or high (*n* = 35)	FAS non BKPyVAN or high (*n* = 170)	PP (*n* = 159)	PP BKPyVAN or high (*n* = 34)	PP non BKPyVAN or high (*n* = 125)	BKPyVAN (*n* = 10)
**BKPyVAN**	10 (4.9)			10 (6.3)			
**Time to BKPyV (d)**	83.5	93		83.5	92.5		83.5
**Male sex**	134 (65.4)	29 (82.3)	105 (61.8)	106 (66.7)	28 (82.4)	78 (62.4)	10 (100.0)
**Age**	54.8	60.3	53.7	56.1	59.8	55.1	57.4
**Weight**	78.2	82.1	77.4	77.7	82.4	76.5	84.5
**Height**	173.1	174.8	172.8	173.0	175.0	172.5	177.8
**BMI**	26.1	26.8	26.0	26.0	26.8	25.8	26.8
**Diabetes**	44 (21.5)	9 (24.3)	35 (20.8)	39 (24.5)	9 (25.0)	30 (24.4)	3 (30.0)
**Primary cause of renal failure**							
Glomerulonephritis	49 (23.9)	7 (18.9)	42 (25.0)	35 (22.0)	7 (19.4)	28 (22.8)	3 (30.0)
Diabetes	37 (18.0)	8 (21.6)	29 (17.3)	33 (20.6)	8 (22.2)	25 (20.3)	3 (30.0)
Cystic/Hereditary/ Congenital	24 (11.7)	4 (10.8)	20 (11.9)	17 (10.7)	4 (11.1)	13 (10.6)	1 (10.0)
Hypertension/Large vessel disease	31 (15.1)	7 (20.0)	24 (14.1)	25 (15.7)	7 (20.6)	18 (14.4)	1 (10.0)
Miscellaneous/unknown	57 (27.8)	8 (22.6)	49 (28.8)	44 (27.7)	7 (20.6)	37 (29.6)	2 (20.0)
Interstitial nephritis/pyelonephritis	6 (2.9)	1 (2.7)	5 (3.0)	4 (2.5)	1 (2.8)	3 (2.4)	0 (0)
Vasculitis/Secondary glomerulonephritis	1 (0.5)	0 (0)	1 (0.6)	1 (0.6)	0 (0)	1 (0.8)	0 (0)
**Simultaneous pancreas tx**	2 (1.0)	1 (2.7)	1 (0.6)	2 (1.3)	1 (2.8)	1 (0.8)	1 (10.0)
**ABO incompatible**	21 (10.2)	4 (10.8)	17 (10.1)	16 (10.1)	4 (11.1)	12 (9.8)	0 (0)
**First tx**	187 (91.2)	32 (91.4)	155 (91.2)	147 (92.5)	31 (91.2)	116 (92.8)	10 (100.0)
**Second tx**	16 (7.8)	3 (8.1)	13 (7.7)	11 (6.9)	3 (8.3)	8 (6.5)	0 (0)
**Third or more tx**	2 (1.0)	0 (0)	2 (1.2)	1 (0.6)	0 (0)	1 (0.8)	0 (0)
**Living donor**	73 (35.6)	10 (27.0)	63 (37.5)	51 (32.1)	10 (27.8)	41 (33.3)	1 (10.0)
**Donor age**	54.4	61.1	53.0	56.4	60.8	55.2	59.4
**Donor male sex**	99 (48.3)	15 (42.3)	84 (49.4)	78 (49.1)	15 (44.1)	63 (50.4)	4 (40.0)
**Recipient CMV+**	138 (67.3)	24 (68.6)	114 (67.1)	107 (67.3)	23 (67.6)	84 (67.2)	5 (50.0)
**Donor CMV+**	149 (72.7)	30 (85.7)	119 (70.0)	115 (72.3)	29 (85.3)	86 (68.8)	8 (80.0)
**CMV mismatch (d+/r-)**	44 (21.5)	10 (27.0)	34 (20.2)	36 (22.6)	10 (27.8)	26 (21.1)	4 (40.0)
**Delayed graft function (d)**	1.9	2.1	1.9	2.0	2.1	2.0	1.9
**Enhanced induction**	31 (15.1)	5 (13.5)	26 (15.5)	23 (14.5)	5 (13.9)	18 (14.6)	1 (10.0)
**Rejection treatment**	48 (23.4)	10 (27.0)	38 (22.6)	40 (25.2)	9 (25.0)	31 (25.2)	5 (50.0)
**SM-resistant rejection treatment**	13 (6.3)	1 (2.7)	12 (7.1)	11 (6.9)	1 (2.8)	10 (8.1)	1 (10.0)
**Graft loss or death**	10 (4.9)	1 (2.7)	9 (5.4)	10 (6.3)	1 (2.8)	9 (7.3)	1 (10.0)
**Death**	3 (1.5)	0 (0)	3 (1.8)	3 (1.9)	0 (0)	3 (2.4)	0 (0)

Demographics and clinical characteristics of kidney- and kidney/pancreas graft recipients with BKPyVAN or high-level DNAemia compared to those with low-level DNAemia or BKPyV negative graft recipients. The table also shows a breakdown of the Full analysis set (FAS) and those who followed the study Per protocol (PP). The number of patients with rejection treatment in this table is both prior to, concurrent with, and after developing BKPyVAN.

### Immunosuppressive treatment

#### Induction therapy

In the FAS, a total of 200 patients (97.6%) received methylprednisolone and basiliximab as induction therapy. The remaining 5 patients (2.4%) received methylprednisolone alone. Also, 31 patients received enhanced induction, mostly in addition to SOC. Of these, 21 were AB0 incompatible.

#### Maintenance therapy

Calcineurin inhibitor (tacrolimus) were given as maintenance immunosuppression to 202 out of all 205 patients (98.5%) while belatacept was given to 3 patients (1.5%). In addition, 202 patients (98.5%) received MMF. All patients (100%) received prednisolone in tapering doses.

### BKPyVAN and BKPyV DNAemia

In total, 10 patients developed biopsy confirmed BKPyVAN resulting in an incidence of 4.9%. Median time to BKPyVAN was 2.7 months (1.6–14.9 months). All but one (90.0%) were diagnosed within one year of transplantation. Thirty-three out of 205 (16.1%) patients met criteria for high-level BKPyV DNAemia, including 8 of the patients with BKPyVAN. Nineteen patients were positive in plasma, but at a low level, and 28 patients were positive only in urine (Table 2).

**Table 2:** Patients with BKPyV DNA in plasma and/or urine

	FAS (*n* = 205)	PP (*n* = 159)
a) High-level ≥ 10,000 copies/mL	33 (16.1)	32 (20.1)
b) Low-level 400 − 10,000 copies/mL	19 (9.2)	13 (8.2)
c) Urine only	28 (13.7)	22 (13.8)
	80 (39.0)	67 (42.1)

**Fig. 2:**
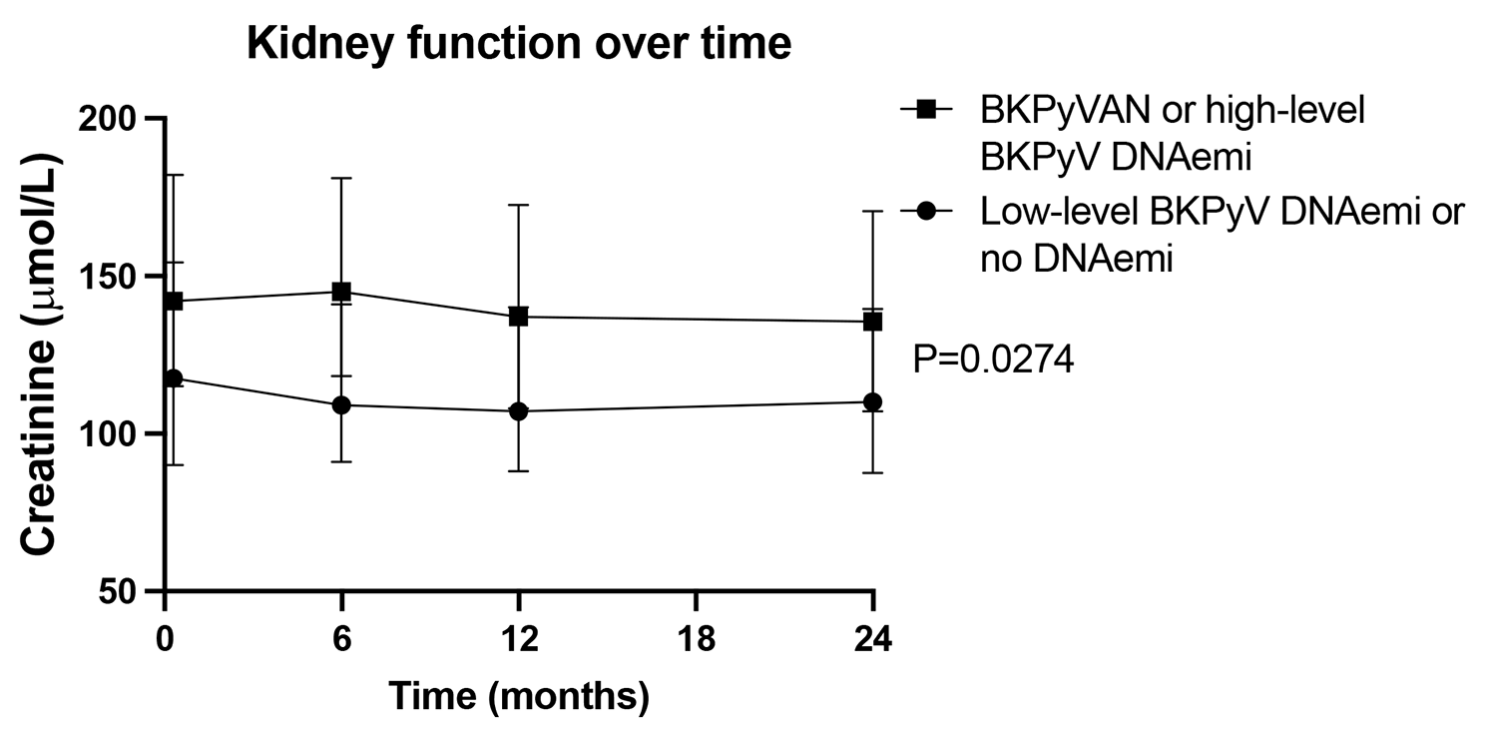
Kidney function over time. Median with interquartile range of creatinine levels in patients with BKPyVAN or high-level BKPyV DNAemia compared to patients with low-level BKPyV DNAemia or no DNAemia. A *p*-value of < 0.05 was considered statistically significant

In four of the patients with high-level BKPyV DNAemia a biopsy was performed but BKPyVAN diagnostics was negative. In the remaining patients, renal function improved after reduction of immunosuppressants before a biopsy was considered necessary. Altogether, 35 (17.1%) developed BKPyVAN or high-level BKPyV DNAemia. The median time until diagnosis was 3.1 months (1.6–17.7) and 31 patients (88.6%) were diagnosed within one year of transplantation. Serum creatinine levels were significantly higher in the BKPyVAN or high level BKPyV DNAemia group, already from start of follow-up (Fig. 2).

### Risk factors for BKPyVAN or high-level BKPyV DNAemia

In the multivariable logistic regression model for the FAS, male sex was identified as a significant risk factor for developing BKPyVAN or high-level BKPyV DNAemia (OR 2.85, *p* = 0.025) along with age (OR 1.03 per year, *p* = 0.020). However, when limiting the analysis to the PP subset, only male sex remained statistically significant. Neither AB0 incompatibility nor rejection treatment was recognized as statistically significant risk factors for development of BKPyVAN or high-level of BKPyV DNA in plasma (Table 3).

**Table 3:** Risk factors for BKPyVAN or high-level BKPyV DNAemia

		FAS			PP	
	OR	95% CI	*p*-value	OR	95% CI	*p*-value
Male sex	2.85	1.14–7.12	0.025*	2.71	1.06–6.96	0.038*
ABO incompability	1.46	0.46–4.66	0.52	1.52	0.45–5.15	0.50
Age (per year)	1.03	1.01–1.06	0.020*	1.02	0.99-1-05	0.15
Rejection treatment	0.51	0.19–1.41	0.20	0.40	0.13–1.19	0.10

OR - odds ratio; CI - confidence interval. *A *p*-value of < 0.05 was considered statistically significant.

### Genotypes

Samples from 76 of the 80 patients that were DNA positive in plasma and/or urine were successfully genotyped. Of these, the most common subtypes were Ib2 and IVc2, in 65.8% and 26.3% patients respectively. Other subtypes identified included Ia (1.3%), Ib1 (3.9%) and 1c (2.6%). Restricting the analysis to patients who were positive in plasma yielded a similar result, with 74.0% genotype I samples and 26.0% genotype IV samples in the FAS. Twenty-eight patients tested positive solely in urine, of whom 26 could be genotyped. Of these, the majority belonged to subtype Ib2 (69.2%) or IVc2 (26.9%). The genotypes and subtypes are presented in Table 4.

**Table 4:** The distribution of genotypes and subtypes in the FAS

Subgroup	Ia	Ib1	Ib2	Ic	IVc2	Unknown	Total
BKPyVAN	1		7		2		10
a) High-level ≥ 10,000 copies/mL			22	2	9		33
b) Low-level 400 − 10,000 copies/mL	1	2	10		4	2	19
c) Urine only		1	18		7	2	28
Total	1	3	50	2	20	4	80

The data available do not indicate any substantial differences in subtype distribution in relation to BKPyVAN diagnosis or viral load.

### Virological course and outcome of BKPyVAN

**Fig. 3:**
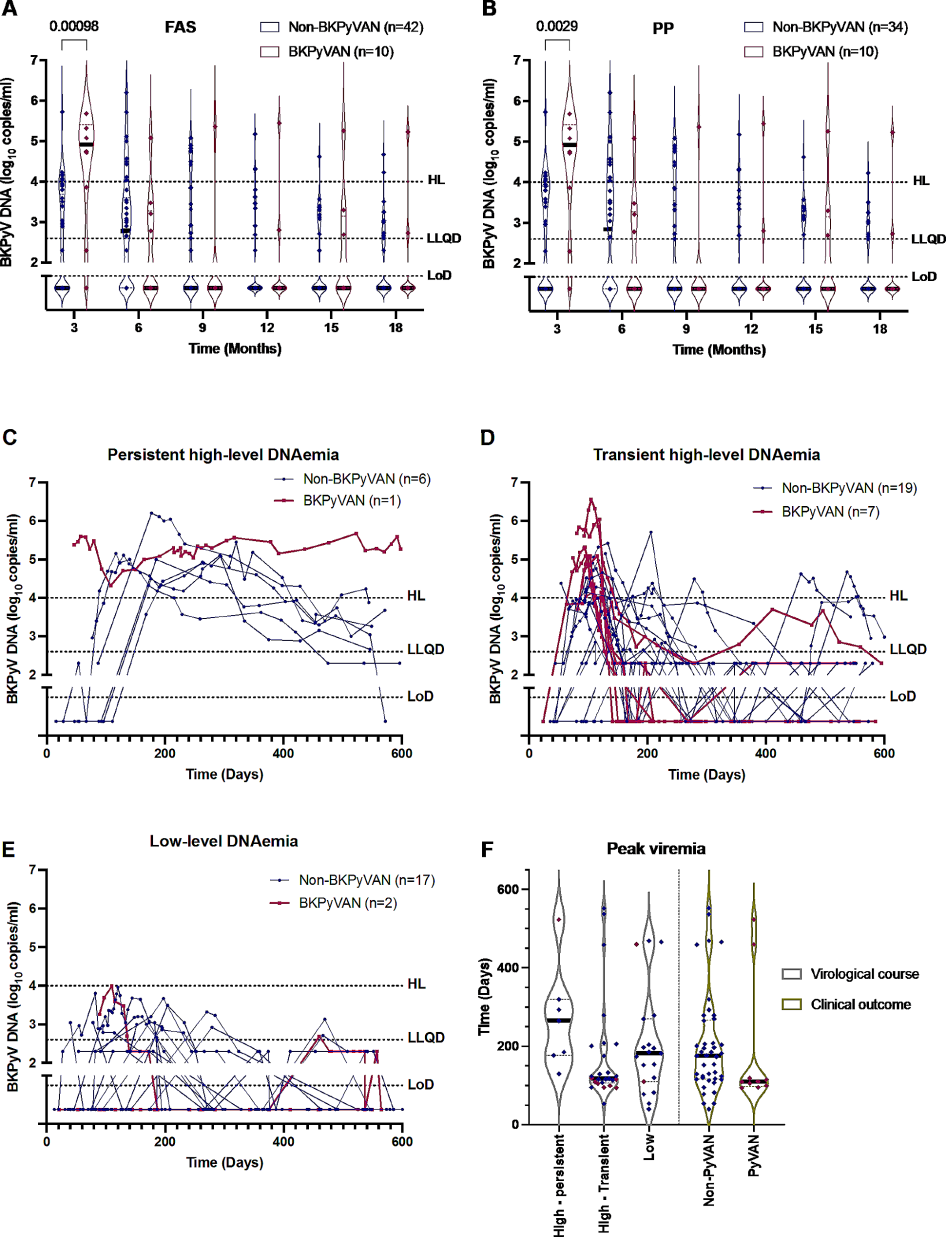
BKPyV DNA levels in plasma for viremic patients. **A**. BKPyV DNA levels at six prescheduled time points in the FAS cohort, where non-BKPyVAN (*n* = 42, blue) and BKPyVAN (*n* = 10, red) are indicated. **B**. BKPyV DNA levels at six prescheduled time points in the PP cohort, where non-BKPyVAN (*n* = 34, blue) and BKPyVAN (*n* = 10, red) are indicated. **C**. Virological course of seven patients with high-level of BKPyV DNAemia for > 3 months. **D**. Virological course of 26 patients with high-level of BKPyV DNAemia for < 3 months. **E**. Virological course of 19 patients with low-level of BKPyV DNAemia. **F.** Time when peak BKPyV DNAemia occurred for the virological courses and endpoint diagnosis. Abbreviations: High-level DNAemia (HL), Lowest Level of Quantitative Determination (LLQD) and Limit of Detection (LoD) are indicated by dotted lines, whereas median values are indicated by black lines. Statistical significance was determined by multiple Mann-Whitney U tests with Holm-Sidak correction and is indicated on top when observed

All ten subjects with BKPyVAN tested positive for BKPyV DNA in their plasma and there was an overall difference in virologic course between BKPyVAN and non-BKPyVAN subjects (*p* < 0.001). The correlation of the virological course and BKPyVAN diagnosis was explored by several approaches. First, we compared the incidence of BKPyVAN with non-BKPyVAN using the six pre-specified screening points taken every three months following transplantation. Whereas BKPyV DNA was significantly higher three months post transplantation in both the FAS and PP cohorts, no such correlation was found at any of the following screening points (Fig. 3A-B). To further examine the viral kinetics, the BKPyV-positive patients with high-level DNAemia, in the FAS cohort, were classified based on whether this condition lasted for more than three months or not (Fig. 3C-E). Whereas some cases of BKPyVAN were found in the categories of persistent high-level DNAemia (1/7 patients) and low-level DNAemia (2/19 patients), the majority of BKPyVAN cases were found in the category of transient high-level DNAemia (7/26 patients; Table 5). No cases of BKPyVAN were found in BKPyV negative patients or with BKPyV DNA in urine only. Analysis of the maximum levels of BKPyV DNA also showed that peak DNAemia occurred considerably earlier for patients with transient high DNAemia and BKPyVAN (Fig. 3F).

A comparison of each category against all the others combined revealed a significant difference for transient, high-level DNAemia and BKPyV negative patients (*p* < 0.0001, Table 5). No significant difference was seen for the groups persistent, high-level DNAemia, low-level DNAemia or patients positive only in urine.

**Table 5:** Virologic course and endpoint diagnosis

BKPyV load	FAS (*n* = 205)	BKPyVAN (*n* = 10)	Non-BKPyVAN (*n* = 195)	OR	95% CI	*p*-value
High DNAemia - Persistent (%)	7	1 (14)	6 (86)	3.5	0.28-28	0.30
High DNAemia - Transient (%)	26	7 (27)	19 (73)	22	5.1–79	< 0.0001*
Low DNAemia (%)	19	2 (11)	17 (89)	2.6	0.52-13	0.23
Urine only (%)	28	0 (0)	28 (100)	0.0	0.0-2.2	0.36
BKPyV negative (%)	125	0 (0)	125 (100)	0.0	0.0-0.2	< 0.0001*

Comparison of virological course and outcome of BKPyVAN by Fisher’s exact test. A *p*-value of < 0.05 was considered significant.

Differences in virological course in relation to BKPyVAN diagnosis was investigated further by pair-wise comparison of the sub-groups (Table 6).

**Table 6:** Virological course in relation to BKPyVAN

	1) Persistent	2) Transient	3) Low	4) Urine only	5) Negative
1) Persistent	x	0.81	1.00	0.33	0.13
2) Transient	0.65	x	0.38	0.019*	< 0.0001*
3) Low	1.00	0.26	x	0.32	0.055
4) Urine only	0.20	0.0037*	0.16	x	1.00
5) Negative	0.053	< 0.0001*	0.017*	1.00	x

Pairwise Fisher´s exact test. *P*-values below the diagonal are unadjusted and *p*-values above the diagonal are multiplicity adjusted by controlling the False Discovery Rate (FDR) *A *p*-value of < 0.05 was considered statistically significant.

Transient DNAemia was statistically associated with BKPyVAN compared to both DNA negative patients and patients with DNA in urine only.

## Discussion

The incidence of BKPyVAN in this study was 4.9%, of which 4.4% presented within 12 months, which is in line with other studies where BKPyV screening has been applied [31, 32]. In comparison, the 12-month cumulative incidence in a previous retrospective study, from the time before the introduction of screening, at our center was 3.7% [25]. The apparently limited effect of the screening program might be due to previous under-diagnosis of BKPyVAN and delayed diagnosis of BKPyV-related adverse outcomes after transplantation. In the analysis of risk factors, male sex and older age were significantly associated with our composite endpoint of BKPyVAN and/or high level BKPyV DNAemia, but not AB0 incompability or rejection treatment. Male sex has previously been shown to be a risk factor for BKPyV DNAemia and BKPyVAN [12, 25, 33–35]. Although the mechanism for this effect is not known, several explanations have been suggested, including anatomical, pharmacokinetical/pharmacodynamical and genetic factors [25].

The distribution of genotypes with 73.7% of genotype I and 26.3% of genotype IV is consistent with what would be expected in a European setting, with a slightly higher proportion of genotype IV in the northern and eastern parts of Europe [20]. Our findings are in line with previous work by Wunderink et al. who did not find any association between BKPyV genotype and risk of BKPyV DNAemia or BKPyVAN [24]. In contrast to earlier studies [23, 36] analysis of BKPyV genotypes did not give any significant association with clinically relevant BKPyV infection in our study. Since earlier PCR-detection methods for BKPyV DNA directed against genotype I have been found to selectively underestimate the levels of genotype IV DNA [26, 37] the clinical interpretation of earlier findings might have been skewed against this genotype at lower loads of viral DNA and thus overestimate its pathogenicity in case of high DNAemia [38].

Analysis of the viral kinetics via time-dependent analysis revealed that BKPyVAN was associated with an early and transient DNAemia characterized by high-levels already at the earliest time point. Although the point estimate indicated a potential overrepresentation also of persistent DNAemia in the BKPyVAN group, hypothesis testing in this small sub-group of patients failed to show statistical significance. During the 18-month follow-up period, only one patient with sustained levels of high DNAemia developed BKPyVAN (Fig. 3C), this patient had impaired humoral immunity and low B cell counts. At the time we began our screening program of renal transplant recipients, the recommended interval for BKPyV surveillance was plasma sampling every 3 months after transplantation [39]. However, according to the present guidelines from the American Society of Transplantation (AST) all KT recipients should be screened for BKPyV DNAemia monthly until month 9, and then every 3 months until 2 years after transplantation [14]. It is therefore advisable to start screening earlier and current guidelines have been adapted accordingly. Alternatively, the threshold for the risk of developing clinically relevant BKPyV reactivation, 10,000 copies/mL in plasma, might be too high. In line with this, Hassan S et al. have previously demonstrated that a threshold of ≥10,000 copies/mL underestimates BKPyVAN cases [40]. Due to similar observations, as well as the fact that 10–30% of biopsies are false negative, AST has revised its current recommendations, and now advocates diagnosing probable or presumptive BKPyVAN entirely on DNAemia, with probable BKPyVAN at levels less than 10,000 copies/mL if they persist for more than 3 weeks [14]. Given the recent development of an international standard for quantitative detection of BKPyVAN DNA [41], future harmonization of the quantification techniques should contribute to further improvement of clinical intervention cut-off criteria.

The time-dependent association between BKPyV DNA levels, level and type of immunosuppression and risk of BKPyVAN remains to be fully entangled. Given that interventions are often initiated already at an early stage of plasma BKPyV reactivation, the onward DNA level dynamics could be biased in relation to the clinical outcome in a purely observational setting that might cause the apparent paradox in our dataset where patients with BKPyVAN diagnosis present with lower BKPyV DNA levels during the following months. Alternatively, since activation of cell mediated immunity is not only essential for controlling viral replication but also induces virus-induced pathology by direct and by-stander killing, it cannot be excluded that vigorous activation of cytotoxic T cells (CTL) upon withdrawal of immune suppressants also contributes to increased damage of the kidneys. To further elucidate this question, a randomized trial comparing different DNA level cut-offs for intervention using standardized PCR assay together with predefined time-points for biopsies and CTL responses would be highly informative.

The strength of our study is the prospective study design with frequent sampling at pre-selected time points that allows proper categorization based on viral kinetics and an unbiased collection of a representative data set. Limitations include a lack of BKPyV data at early time points less than three months post transplantation and the fact that the study was conducted in only one center with a relatively small sample size.

## Conclusions

We found that male sex and increasing age, but not AB0 incompability and rejection treatment, are significant risk factors for developing BKPyVAN or BKPyV DNAemia. BKPyVAN is associated with increased viral load shortly after transplantation and with transient, high-level DNAemia but not with genotype. The introduction of a BKPyV screening program has not reduced the incidence but is likely to detect more cases and at earlier time points, which can hopefully reduce the risk of permanent allograft failure or even graft loss.

### Electronic supplementary material

Below is the link to the electronic supplementary material.

Supplementary Material 1

## Data Availability

The datasets used and/or analyzed during the current study are available from the corresponding author on reasonable request.

## Abbreviations

BKPyV: BK Polyomavirus
BKPyVAN: BK Polyomavirus–associated nephropathy
CTL: Cytotoxic T cells
FAS: Full analysis set
FDR: False discovery rate
IVIg: Intravenous immunoglobulin
MMF: Mycophenolate mofetil
PCR: Polymerase chain reaction
PP: Per protocol
SOC: Standard of care
