# Supplementary material for: The risk factors associated with post-transplantation BKPyV nephropathy and BKPyV DNAemia: a prospective study in kidney transplant recipients
Source: BMC Infect Dis. 2024 Feb 22;24:245. doi: 10.1186/s12879-024-09093-7 (PMC10885533; doi:10.1186/s12879-024-09093-7)
Supplement: Supplementary file 1 — Supplementary Material 1 [file 12879_2024_9093_MOESM1_ESM.docx]

**Supplementary table 1:** Primers used for BKPyV quantitative PCR and genotyping.

| **Primers** | **Sequence 5’-3’** | **Position^1^** | **Product size** | **Final concentration** |
| --- | --- | --- | --- | --- |
| BK-F1 | AATGCCTTAATCTAAGCTGACATAG | 4793-4817 | 78 bp | 240 nM |
| BK-FA | AATGTCTTAATCTTAGCTGACACAG | 4793-4817 |  | 240 nM |
| BK-R1 | GCAAGGAATGGCCTATTTGTTCCAAA | 4845-4870 |  | 240 nM |
| BK-RA | GCAAGGAWTGGCCTATTTGTTCCAAA | 4845-4870 |  | 240 nM |
| BK-PB | YY^2^-TGCAAGGGCARTGCACWGAAGGCT-BHQ1^3^ | 4820-4043 |  | 200 nM |
| 1466_F1  2331_R1 | CCCAGGAGGTGCTAATCAAA  TTTACAHAGAGGCCCCACAC | 1466-1485  2312-2331 | 866 bp | 400 nM  400 nM |
| 1528_F2  2270_R2 | ACGGGACTGTAACACCTGCT  TGAAGTACTGGSGGAACATTTTC | 1528-1547  2248-2270 | 743 bp | 400 nM  400 nM |

1. Dunlop numbering
2. Yakima yellow
3. Black hole quencher 1
